# Molecular Simulation Study on the Aging Mechanism of NEPE Propellant Matrix

**DOI:** 10.3390/molecules28041792

**Published:** 2023-02-14

**Authors:** Lingze Kong, Kehai Dong, Yanhui Tang, Chuanlu Yang, Yundong Xiao

**Affiliations:** 1 Department of Aircraft Engineering, Naval Aviation University, Yantai 264001, China; 2 School of Physics and Optoelectronic Engineering, Ludong University, Yantai 264001, China

**Keywords:** molecular simulation, NEPE, PEG, TDI, DFT, VTST, solid propellant

## Abstract

Polyethylene glycols (PEG) and toluene diisocyanate (TDI) are often used as the main components of binders and curing agents in solid propellants, and their aging is an important issue in the storage and use of propellants. To study the aging behavior and aging mechanism of nitrate ester plasticized polyether propellant (NEPE) matrix during storage, the transition states of aging reactions of binder and curing agent were optimized at the (U)B3LYP/6-311G(d,p) level of theory, and the rate coefficients over the temperature range of 298–1000 K were calculated by CVT theory. The results showed that there were five kinds of aging reactions for binder, which included decomposition, nitration, H abstraction, oxidation, and crosslinking reactions. Among them, theenergy barriers of oxidation and H abstraction reactions were relatively low (79.3–91.2 kJ·mol^−1^) and the main reaction types of binder aging. The main aging reaction of curing agent was decomposition. Compared with the aging reactions of binder, the energy barriers of curing agent are higher (196.6–282.7 kJ·mol^−1^) and the reaction is more difficult to occur. By comparing the energy barriers and rate constants of different reactions, it is found that the aging of NEPE propellant matrix can be divided into two stages. In the first stage, the propellant matrix mainly undergoes H abstraction and oxidation reaction, and as the reaction proceeds, the products crosslink to form -O-O-, -C-C-, and -C-O-C- bonds. At this time, the long chain molecules of the propellant matrix crosslink, and the molecular weight increases. This stage corresponds to the rising stage of mechanical properties in the aging process of the propellant. In the second stage, the propellant matrix mainly undergoes decomposition and nitration, resulting in degradation, the reduction of molecular weights, and the appearance of holes and microcracks in the matrix. This stage corresponds to the decline of mechanical properties in the aging process of the propellant. The above simulation results are in good agreement with the aging experimental phenomena, revealing the microscopic mechanism of the changes in the macroscopic properties of NEPE propellant during the aging process, and providing a theoretical basis for the related research on the aging properties and anti-aging technology of NEPE propellant.

## 1. Introduction

Polyethylene glycol (PEG) is often used as the main component of nitrate ester plasticized polyether propellant (NEPE) matrix together with curing agents, such as toluene diisocyanate (TDI) and isophorone diisocyanate (IPDI), because of its good physical and chemical stability, high enthalpy of formation, and gas generation. During the storage of propellant, the weak points, such as -C-O-C- and C-N bonds, in the chemical structure of the binder and curing agent are prone to undergo chemical changes under the action of environmental factors (heat and oxygen), which, in turn, affect the aging performance of the solid propellant [1]. Thus, it is clear that in-depth research on the aging behavior of NEPE solid propellant matrix has important guiding significance for the long-term safe storage of propellants.

At present, much literature has reported the aging behavior of NEPE propellant matrix. The results of thermal decomposition of a NEPE propellant matrix shows that a series of reactions, such as chain breaking and oxidation, will occur between binder and curing agent during thermal degradation, generating products, such as olefins, alcohols, aldehydes, and ketones, and releasing a large amount of gas [2,3,4,5,6,7]. Based on the above research results, Zhang et al. [8,9,10] have concluded that the irregular breakage of molecular chains is the main route of PEG thermal decomposition, which is also an important feature of NEPE propellant aging. Ravey et al. [11,12,13,14,15] have analyzed the thermal degradation steps and reaction mechanism of urethane bonds in polyurethane in the range of 200–250 °C through thermal volatilization testing and summarized three primary thermal degradation reaction routes for urethane. In addition, Li et al. [16] have found that PEG also interacts with the decomposition products of oxidizers and plasticizers in NEPE propellants, allowing oxidation and H abstraction reactions to accelerate PEG aging. Yang et al. [17,18,19] have investigated the correlation between PEG aging behavior and of NEPE propellant mechanical properties and found that molecular structural changes (crosslinking and degradation) of NEPE propellant binder were internal causes for changes in mechanical properties. The above studies have generally used experiments combined with analytical instruments to study the main factors affecting propellant matrix aging, its aging process, and change trends of aging performance. These studies are generally costly, long-term, and have many limitations, such that one can only speculate regarding the aging reactions of the matrix through experimental phenomena and results and cannot deeply analyze the aging reaction mechanism. In recent years, with the rapid development of computational chemistry, molecular simulation has become a powerful tool for revealing microscopic chemical reaction processes and reaction mechanisms, and predicting material properties. Fan et al. [20] have simulated the nitration reaction of NO_2_ with PEG using molecular simulation software. Based on density functional theory (DFT), Pei et al. [21] have calculated the energy barriers and reaction rate constants of degradation, nitration, and cyclization of PEG with different polymerization degrees at the 6-31G(d,p) level and obtained results with good experimental correlation. Wu et al. [22] have calculated the bond-dissociation energy (BDE) in simplified structures of the network model formed by HTPB and TDI using quantum chemical methods and analyzed the relationship between bond energy and aging decomposition. However, studies on the aging behavior of NEPE propellant matrix and its interactions with other components in the propellant still rely on experimental means, and relevant theoretical and kinetic studies have rarely been reported.

With the goal to explore the aging behavior of NEPE propellant matrix and reveal the reaction mechanisms and types of chemical reactions that occur between binder and curing agent during the matrix aging process, in this study, density functional theory (DFT) was used to study PEG and TDI at the 6-311G(d,p) level. First, molecular simulations of aging reaction types and mechanisms of PEG and a simplified molecular model of PEG-TDI were carried out and the energy barriers and rate constants of each aging reaction further were calculated. Finally, the energy barriers and rate constants of different aging reactions were compared and analyzed to obtain the reaction process and mechanism of the matrix during the aging process of NEPE propellant storage.

## 2. Results and Discussion

### 2.1. Aging Reactions of Binder

According to the calculation results of the dissociation energy required for the reaction of PEG molecules with different degrees of polymerization by Pei et al. [21], the degree of polymerization has little effect on the aging reactions of PEG molecules. PEG where *n* is 2 has been found to best represent the aging state of PEG molecules with different degrees of polymerization. Therefore, to save computing power, the PEG model with a polymerization degree of 2 was chosen here as the object of study for the binder aging reaction. Sun et al. [23,24] have carried out molecular simulations of nitration and H abstraction reactions of PEG by DFT. As the purpose of their study was different from this study, the simulations of nitration and H abstraction reactions had to be recalculated to facilitate comparison of the differences between different aging reactions of the matrix of NEPE propellant. Five main reactions were calculated for the aging of PEG (*n* at 2) at the 6-311G(d,p) level, including decomposition, nitration, oxidation, H abstraction, and crosslinking reactions.

#### 2.1.1. Decomposition Reaction

**Figure 1:**
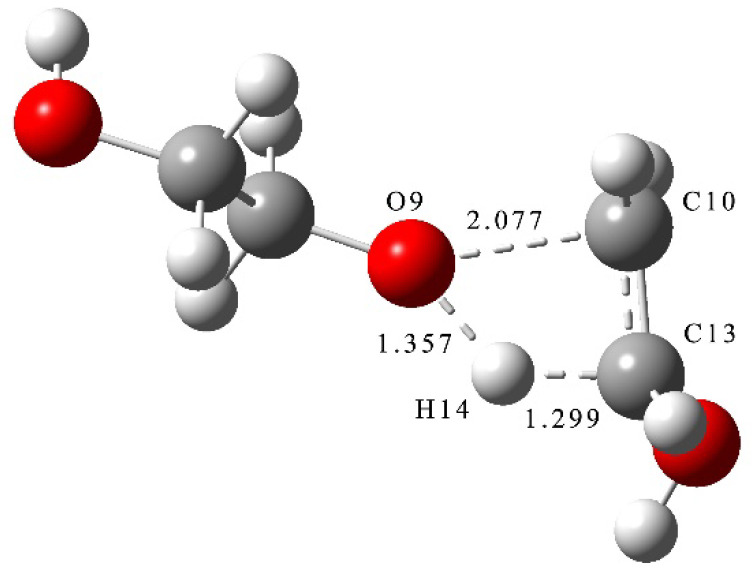
Transition state of decomposition reaction of PEG.

Experimental studies of thermal decomposition have shown that there is a direct breakage of unstable chemical bonds in the PEG molecule leading to PEG decomposition to produce alcohols [7,25]. The equation for the decomposition reaction of the binder was obtained by molecular simulation, as shown in R1:

*HOCH*_2_*CH*_2_*OCH*_2_*CH*_2_*OH* (*PEG*, *n* = 2) → *TS*_1_ → 
*HOCHCH*_2_ (*P*1) + *HOCH*_2_*CH*_2_*OH* (*P*2) (R1)

where *TS*_1_ is the transition state of the decomposition reaction of PEG (Figure 1).

**Figure 2:**
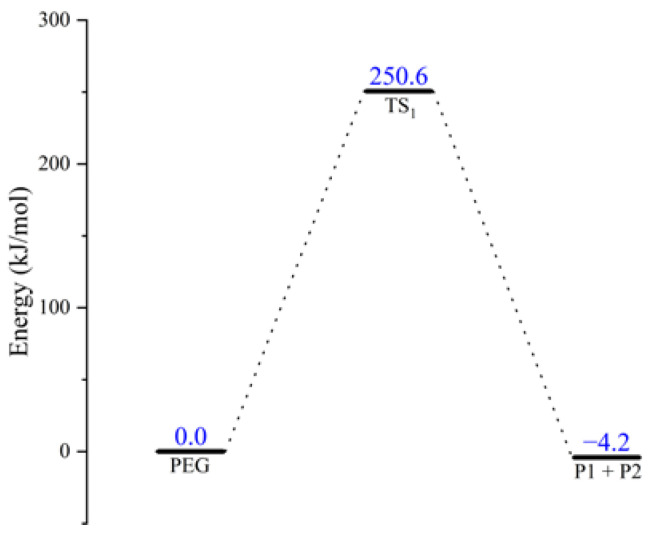
Schematic energy diagram of decomposition reaction of PEG.

**Figure 3:**
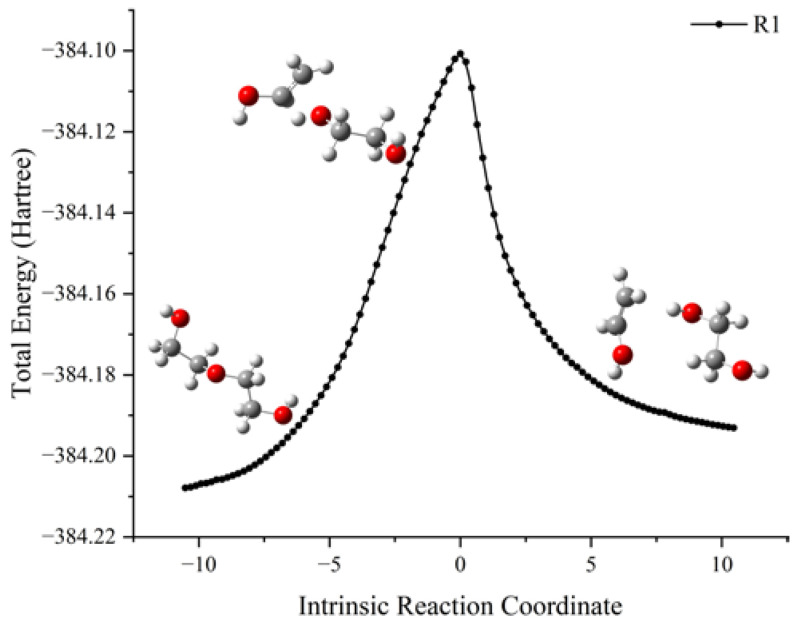
IRC curve of decomposition reaction of PEG.

The schematic energy diagram for PEG decomposition reaction was obtained from molecular simulation (Figure 2). The intrinsic reaction coordinate (IRC) curve that connects the pathway between the reactants and products is shown in Figure 3.

From the results of transition state calculations, at the beginning of the reaction and under the attraction of O9, it was seen that the bond length of C13-H14 gradually increased from 1.10 Å to 1.30 Å in TS_1_. At this time, O9-C10 cleavage occurred by stretching vibrations and, as the reaction continued, the distance between H14 and O9 shrank continuously to form an -OH group. Then, the PEG molecular chain was broken, molecular weight reduced, and an enol produced. Due to the presence of carbon–carbon double bonds in enols, they are relatively unstable and easily converted into carbonyl structures containing stable carbon–oxygen double bonds. The energy calculations of the products and reactants showed that the energy of the products was decreased by 4.2 kJ·mol^−1^ compared with the reactants, indicating that the decomposition reaction was exothermic.

#### 2.1.2. Nitration Reaction

**Figure 4:**
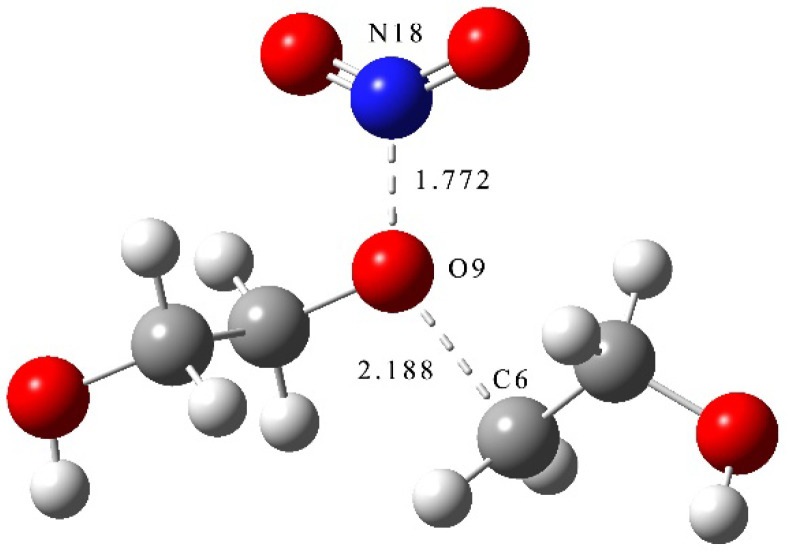
Transition state of nitration reaction of PEG.

The results of thermal decomposition tests conducted by Bohn et al. have shown that the bond energy of the RO-NO_2_ bond in nitrate is low so that the RO-NO_2_ bond can break directly to generate NO_2_. The NO_2_ generated by the RO-NO_2_ bond will then attack the O atom in PEG during long-term storage of NEPE propellant, causing PEG degradation by breaking the chains [8,26,27,28]. The equation for the nitration reaction of the PEG was obtained by molecular simulation, expressed in R2 as:

*OHCH*_2_*CH*_2_*OCH*_2_*CH*_2_*OH* (*PEG*, *n* = 2) + *NO*_2_ → *TS*_2_ → 
*HOCHCH*_2_ (*P*3) + *HOCH*_2_*CH*_2_*ONO*_2_ (*P*4) (R2)

where *TS*_2_ is the transition state of the nitration reaction of PEG (Figure 4).

**Figure 5:**
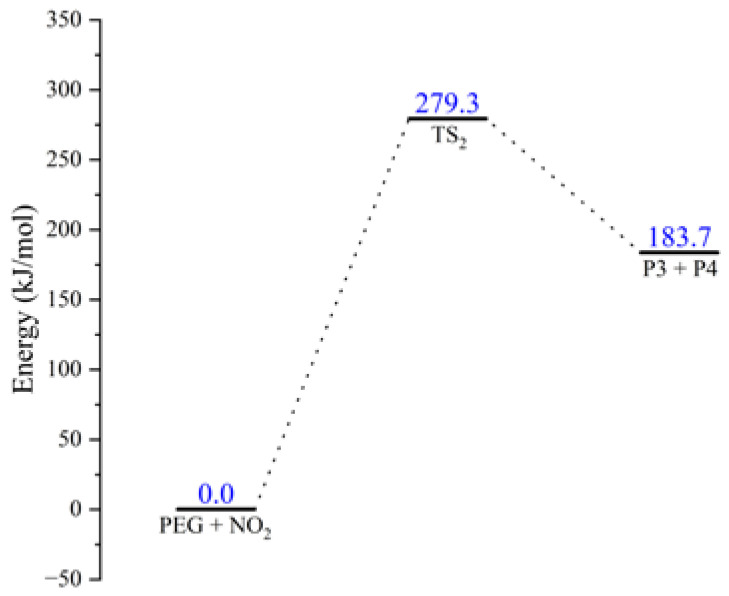
Schematic energy diagram of nitration reaction of PEG.

**Figure 6:**
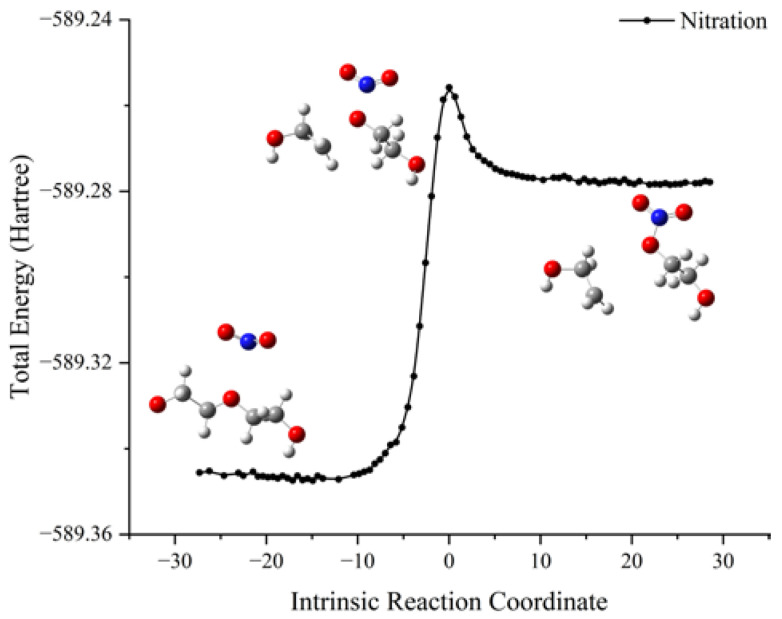
IRC curve of nitration reaction of PEG.

The schematic energy diagram for the nitration reaction of PEG was obtained from molecular simulation (Figure 5). IRC curve that connects the pathway between the reactants and products are shown in Figure 6.

From the results of transition state calculations, it was seen that, at the beginning of the reaction, the distance between the N atom in NO_2_ and O atom in PEG shrank from 1.77 to 1.42 Å in TS_1_ under electrostatic effects. At this time, the C-O bond in the PEG molecule was gradually increased from 1.42 to 2.19 Å by the attraction of NO_2_ and, as the reaction continued, the bond length of N-O gradually shortened, the bond length of C-O gradually increased, and the PEG molecular chain was broken. The energy calculations from the reactants and products showed that the energy of the products increased by 183.7 kJ·mol^−1^ compared with that of the reactants, indicating that the nitration reaction was an endothermic reaction.

#### 2.1.3. H Abstraction Reaction

The results of infrared spectroscopy and nuclear magnetic resonance experiments on the thermal decomposition of PEG by Luo et al. showed that in addition to the decomposition reaction with PEG, the NO_2_ also attacked the H atom attached to the C atom on the PEG molecular chains, causing H abstraction reaction of PEG [29]. The equations for the H abstraction reaction of PEG were obtained by molecular simulation, as expressed in R3-1 and R3-2 as:

*OHCH*_2_*CH*_2_*OCH*_2_*CH*_2_*OH* (*PEG*, *n* = 2) + *NO*_2_ → *TS*_3_ → 
*OHCH*_2_*CH*_2_*OCHCH*_2_*OH* (*P*5) + *ONHO* (*P*6) (R3-1)

**Figure 7:**
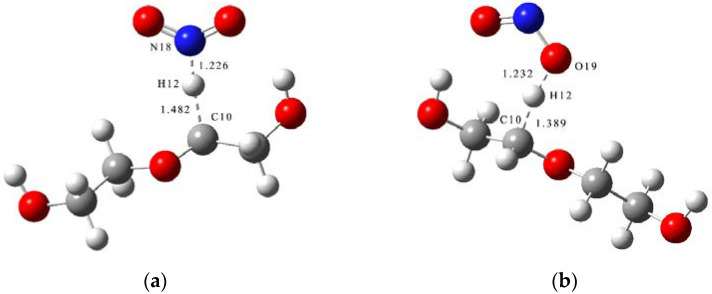
Transition states of H abstraction reaction of PEG. (**a**) TS_3_ and (**b**) TS_4_.

*OHCH*_2_*CH*_2_*OCH*_2_*CH*_2_*OH* (*PEG*, *n* = 2) + *NO*_2_ → *TS*_4_ → 
*OHCH*_2_*CH*_2_*OCHCH*_2_*OH* · (*P*7) + *HONO* (*P*8) (R3-2)

where *TS*_3_ and *TS*_4_ are the transition states of the H abstraction reaction of PEG (Figure 7).

**Figure 8:**
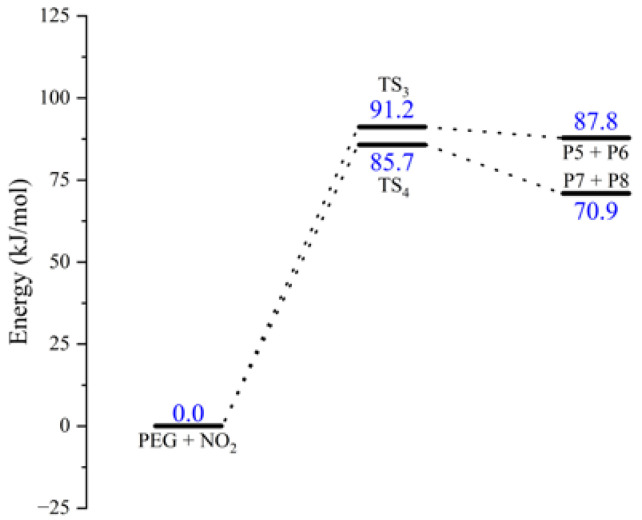
Schematic energy diagram of H abstraction reaction of PEG.

**Figure 9:**
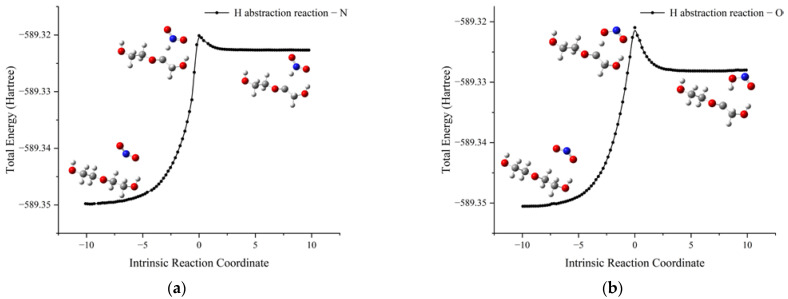
IRC curves of H abstraction reaction of PEG. (**a**) Reaction3-1 and (**b**) Reaction3-2.

The schematic energy diagram for the H abstraction reaction of PEG was obtained from molecular simulation (Figure 8). IRC curves that connect the pathways between the reactants and products are shown in Figure 9.

From the results of transition state calculations, the bond lengths of C-H bonds in the PEG molecular chain at the beginning of the reaction were seen to gradually increase under the attraction of NO_2_ molecules and the distance between the H atom with N or O atom decreased from 3.31 or 2.74 Å in the reactants to 1.23 Å in TS_3_/TS_4_, respectively, forming O-H and N-H bonds, at which time the bond lengths of the C-H bonds reached 1.48 and 1.39 Å, respectively, i.e., breakage occurred. The results of the energy barrier calculations showed that the energy barrier required for the O atom to initiate the H abstraction reaction during the reaction between PEG and NO_2_ was slightly smaller than that required for the N atom to initiate the H abstraction reaction. The main reason for this difference was the different electronegativity and site resistance of the N and O atoms in the NO_2_ molecule [23]. In this reaction, the energy of the products (P5 + P6 and P7 + P8) increased by 87.8 and 70.97 kJ·mol^−1^, respectively, compared with the reactants, indicating that the H abstraction reaction was an endothermic reaction.

#### 2.1.4. Oxidation Reaction

**Figure 10:**
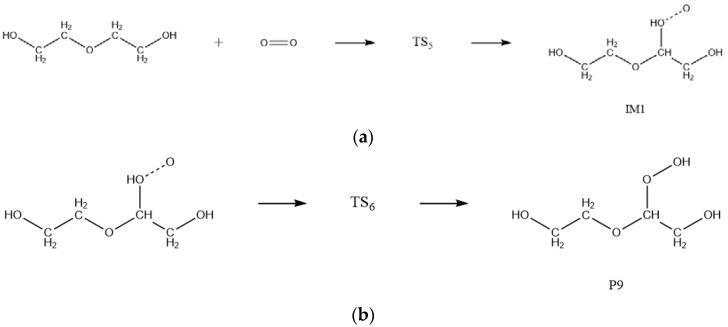
Equations of oxidation reaction of PEG. (**a**) R4-1 and (**b**) R4-2.

**Figure 11:**
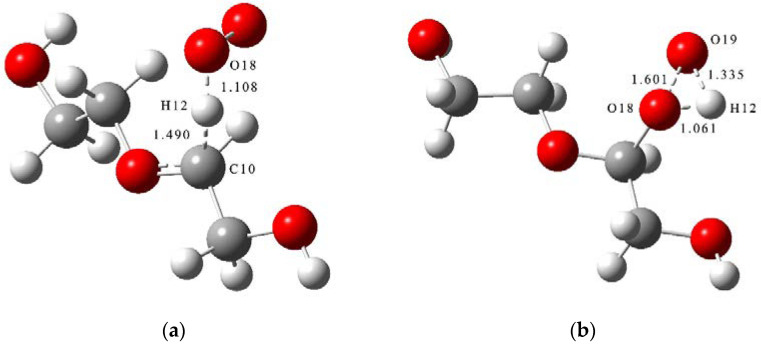
Transition states of oxidation reaction of PEG. (**a**) TS5 and (**b**) TS_6_.

Boldyrev et al. [30,31,32] have found that ammonium perchlorate (AP) will decompose at a certain temperature to release oxygen and other gases. Combined with the results of thermal oxygen aging tests conducted by Han et al. for PEG, it is known that NEPE propellant binder can oxidize with the oxygen released from AP in the propellant during storage [3,6]. The pathways for the oxidation reaction of PEG were obtained by molecular simulation and shown in Figure 10. The transition states of the oxidation reaction of PEG are shown in Figure 11.

**Figure 12:**
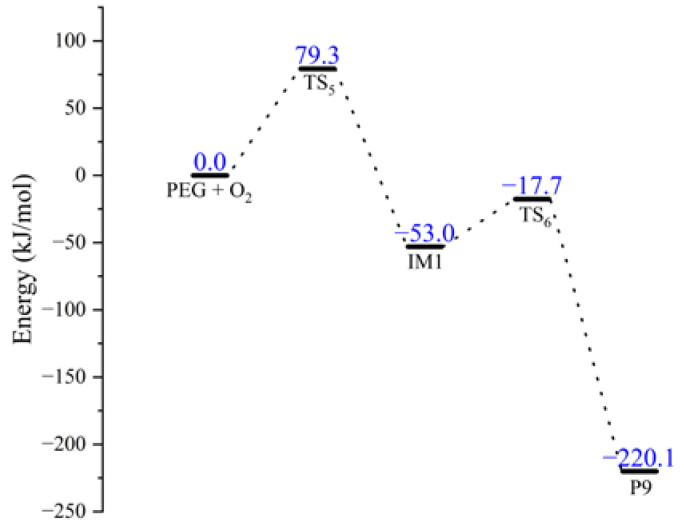
Schematic energy diagram of oxidation reaction of PEG.

**Figure 13:**
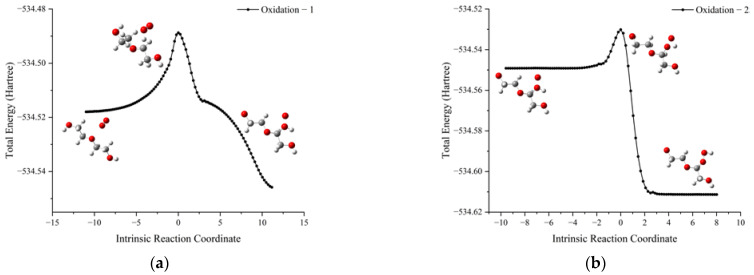
IRC curves of oxidation reaction of PEG. (**a**) Reaction 4-1 and (**b**) Reaction 4-2.

The schematic energy diagram for the oxidation reaction of PEG was obtained from molecular simulation (Figure 12). IRC curves that connect the pathways between the reactants and products are shown in Figure 13.

From the results of transition state calculations, the oxidation was seen to proceed in two steps. At the beginning of the reaction, the H atom in PEG was attracted by O_2_ and gradually moved away from the PEG molecule and the bond length of the C10-H12 bond in the PEG molecule increased from 1.10 to 1.49 Å in TS_5_, followed by the generation of an intermediate (IM1). As the reaction continued, the H12 atom moved closer to O19 and the bond lengths of H12-O18 and H12-O19 bonds changed from 0.98 and 1.99 in IM1 to 1.06 and 1.34 Å in TS_6_, respectively. When reaction 4-1 is completed, peroxide P9 was generated, and because of the unstable peroxygen bond in P9, it easily decomposed into alkoxy radicals (·OR) and hydroxyl radicals (·OH) under the action of environmental factors. From the results of energy barrier calculations, the energy barrier required for the occurrence of R4-1 was seen to be larger than that required for the occurrence of R4-2. This indicated that most of the products would exist in the form of P9 and its products during the PEG oxidation reaction. The energy of the intermediate product (IM1) and product (P9) in this reaction were lower by 53.0 and 167.1 kJ·mol^−1^, respectively, compared to reactants, indicating that the oxidation reaction was exothermic.

#### 2.1.5. Crosslinking Reaction

**Figure 14:**
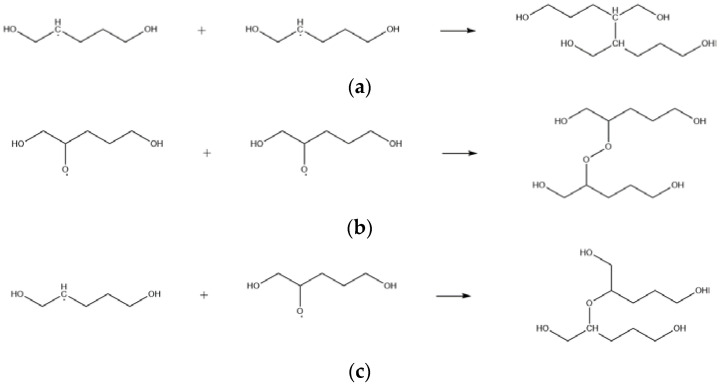
Equations of PEG crosslinking reactions. (**a**) R5-1; (**b**) R5-2; (**c**) R5-3.

Zhao et al. [10,17] have found that the crosslink density of NEPE propellant shows a trend of rising and then decreasing with increased aging time via an accelerated aging test, indicating the existence of crosslinking reactions of the binder in the early stage of aging. The pathways for the crosslinking reactions of PEG were obtained by molecular simulation, shown in Figure 14.

The products of the H abstraction and oxidation reactions contain alkyl and alkoxy radicals, respectively. With increased radical concentration, alkyl and alkoxy radicals can combine in a manner without potential energy barriers to form conformationally stable and low energy -C-C- bonds, -O-O-, and -C-O-C- structures [33], i.e., crosslinking reactions occur, in which an irregular network structure is formed between molecules, matrix molecular weight increases sharply, and crosslinking density and gel percentage rise accordingly [17].

**Figure 15:**
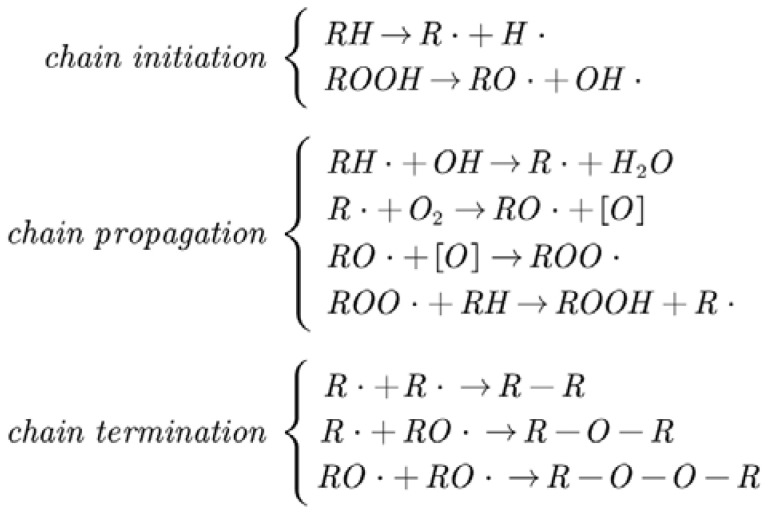
Chain reaction process of binder.

According to the reaction mechanism of H abstraction, oxidation, and crosslinking reactions of binder, it can be found that the reaction process of these three reactions follows the radical chain reaction mechanism consisting of chain initiation, chain propagation, and chain termination. The whole chain reaction process is shown in Figure 15. It can be seen that in the chain initiation stage, the C-H bond in the PEG is attacked by NO_2_ and O_2_ to produce active radicals such as alkyl (R·) and alkoxy (RO·); in the chain propagation stage, the unstable active radicals can react with PEG and O_2_ rapidly to generate new free radicals, leading to the increasing concentration of free radicals in the matrix; in the chain termination stage, the free radicals are combined together in a potential barrier free manner to form macromolecular peroxides, which leads to the crosslinking reaction. As the concentration of free radicals in the matrix continuously decreases, the chain reaction ends.

Since the reaction rate of free radical chain reaction mainly depends on the concentration of active free radicals in the reaction process, the aging reaction of propellant matrix be effectively delayed by preventing the generation of free radicals.

### 2.2. Aging Reactions of Curing Agent

**Figure 16:**
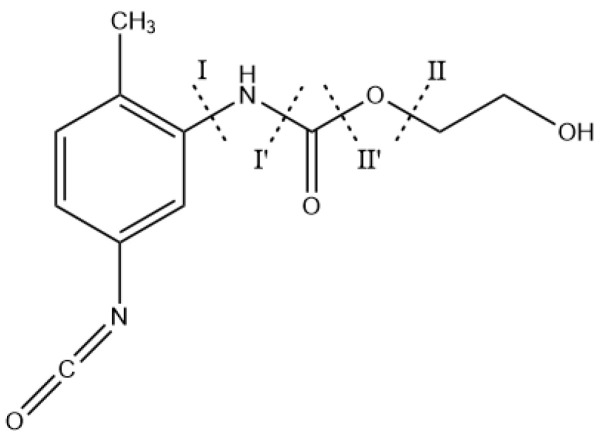
Dissociation position of PT characteristic bonds.

TDI is often used as a curing agent to react with PEG prepolymer to generate PEG-TDI (PT) structures. The aging types of PEG have been calculated and analyzed in Section 3.1. To study the effects of addition of TDI on the aging of propellant matrix, the PT structure has been simplified to the model shown in Figure 16. The results of thermal decomposition experiments conducted by Allan et al. [11,12,13,14,15] have shown that unstable C-N and C-O bonds present in carbamate can generate a large amount of polyol and carbon dioxide (CO_2_) gas during the aging process. To investigate the reaction types and mechanism of the aging reaction of PT molecules, the bond-dissociation energy of the characteristic chemical bonds in PT molecules was first calculated. The calculated BDE of different bonds in PT molecules are shown in Table 1, and the positions of different characteristic chemical bonds in PT molecules are shown in Figure 16.

**Figure 17:**
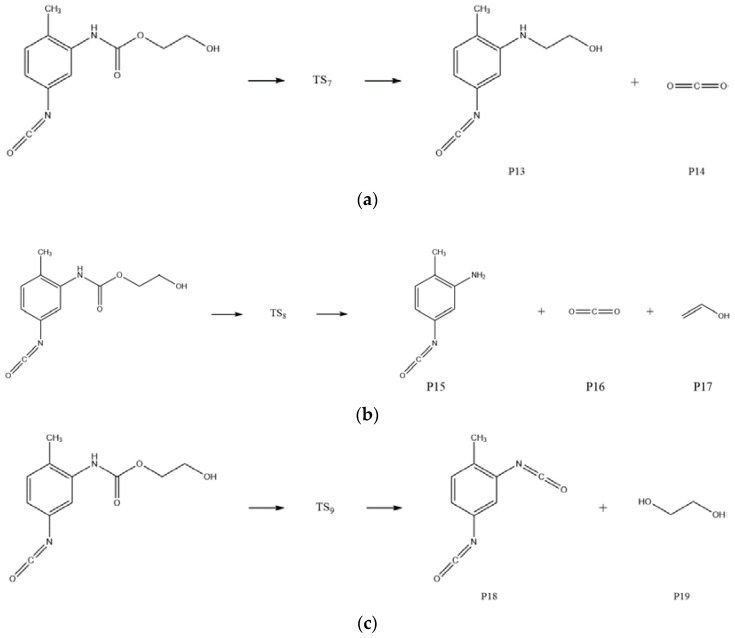
Equations of decomposition reactions of PT. (**a**) R6-1; (**b**) R6-2; (**c**) R6-3.

**Figure 18:**
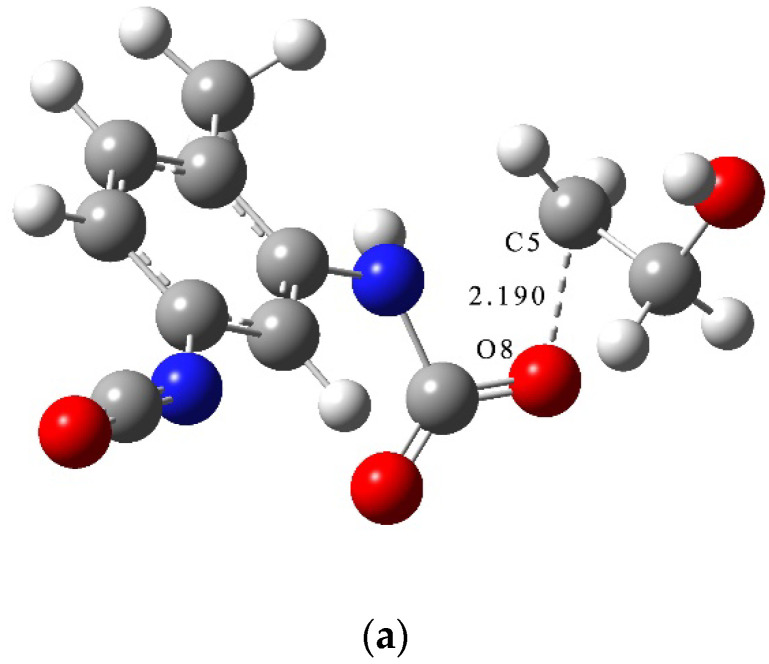

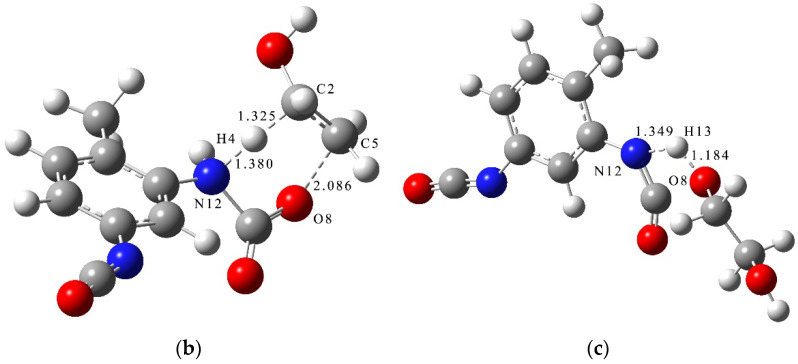
Transition states of decomposition reactions of PT. (**a**) TS_7_; (**b**) TS_8_; (**c**) TS_9_.

From the results of C-N dissociation energy calculations, the bond dissociation energy of the C(Ph)-N bond (C-N bond formed by N connected with C on benzene ring) at the ortho-position was seen to be slightly smaller than that of the bond dissociation energy at the para-position. The dissociation energy of the C(Ph)-N bond (Ⅰ) in the PT molecule had a value which was significantly larger than the dissociation energy of C-N (Ⅰ′) formed by N and C attached to O, indicating that the phenyl ring was conducive to stability of the binder-curing agent structure. From the results of C-O dissociation energy calculations, the C-O bond of Ⅱ was seen to have the smallest bond dissociation energy, which predicted that the chemical bond was easily broken and decomposed during the PT aging process. The results of dissociation energy calculations suggested that homolysis of C-N and C-O bonds in the PT molecule was the main cause of the PT aging reaction. At the level of 6-311G(d,p), the main aging reactions of the PT structure were calculated to be decomposition reactions and there existed three reaction pathways. The reaction pathways corresponding to these reactions are shown in Figure 17. The transition states of the decomposition reaction of PT are shown in Figure 18.

**Figure 19:**
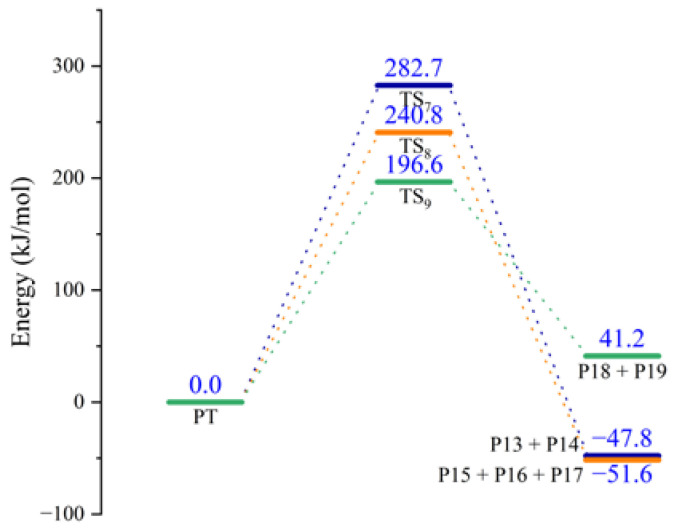
Schematic energy diagram of decomposition reactions of PT.

**Figure 20:**
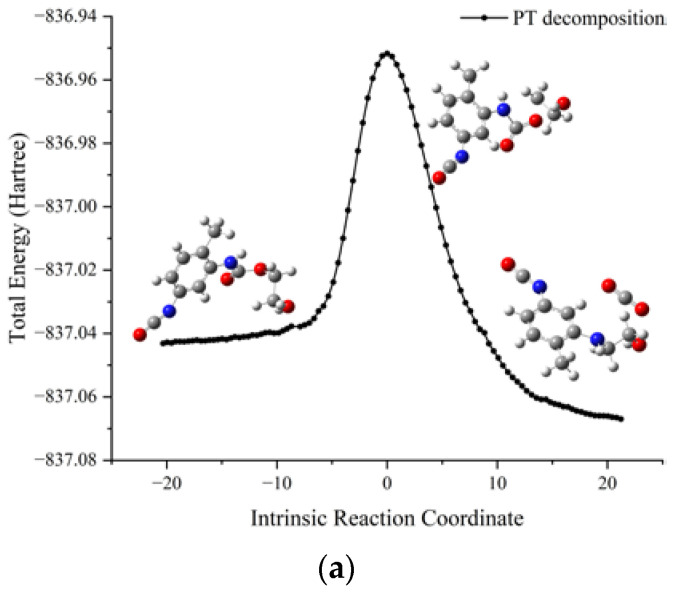

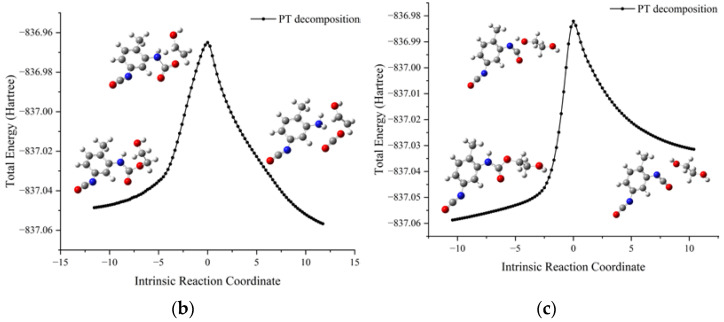
IRC curves of decomposition reaction of PT. (**a**) R6-1; (**b**) R6-2; (**c**) R6-2.

The schematic energy diagram for the decomposition reaction of PT was obtained from molecular simulation (Figure 19). IRC curves that connect the pathways between the reactants and products are shown in Figure 20.

From the results of transition state calculations, the aging reaction process of PT was seen to be able to be divided into three main stages. First, the C-O bond with the smallest bond dissociation energy in the PT molecule was the first to break to form free radicals. Then, C(O)-N breaks to generate free radicals RNH· and CO_2_. Eventually RNHC(O)O·, RNH·, and other unstable radicals might continue to react with other radicals and active components in the aging process to produce alcohols and benzidine. Among the three reactions, the PT structures in R6-2 and R6-3 both underwent degradation and, although the amino radicals generated in R6-1 still maintained the NH group after recombination with alkyl radicals, the results showed that hydrogen bonds related to NH were easily dissociated at 40–100 °C [34,35,36]. The dissociation of PT during thermal aging, together with the local holes and microcracks formed by the accumulation of CO_2_ in NEPE matrix, destroyed the curing network of NEPE propellant matrix, which led to the continuous decline of the mechanical properties of propellant with increased aging time [18,37]. From the results of reaction energy barrier calculations, the energy of the products of R6-1 and R6-2 were seen to be 47.8 and 51.6 kJ·mol^−1^ lower than that of the reactants, respectively, indicating that R6-1 and R6-2 were exothermic reactions. The energy of the products of R6-3 was 41.2 kJ·mol^−1^ higher than that of the reactants, indicating that R6-3 was an endothermic reaction and its decomposition to PEG and TDI is less likely to occur under normal atmospheric temperature.

### 2.3. Analysis of Matrix Aging Mechanism

**Figure 21:**
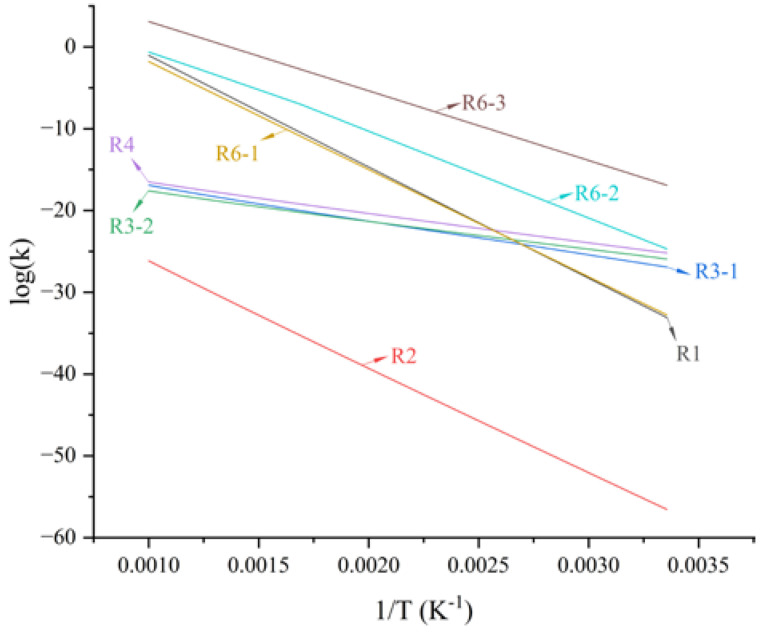
Reaction rate constants of different reactions.

**Figure 22:**
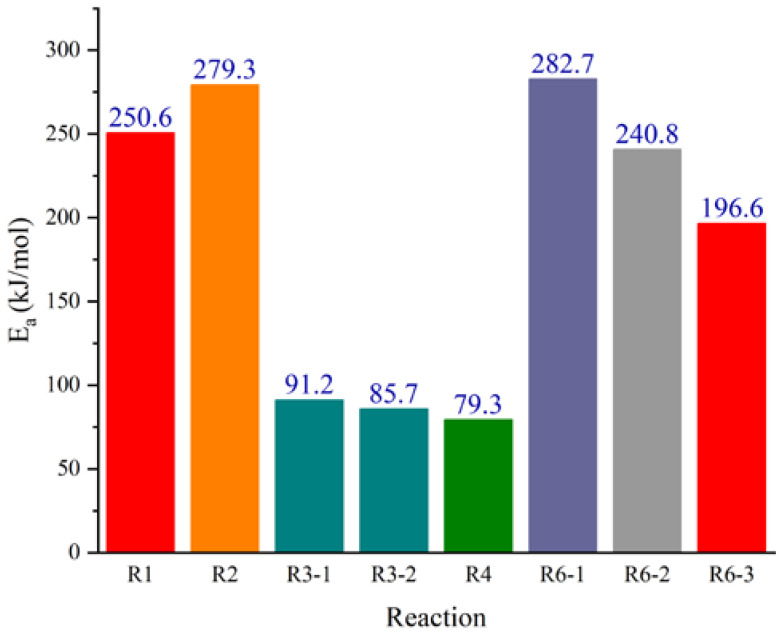
Energy barriers of different reactions.

To further reveal the aging behavior and mechanism of the matrix in NEPE propellant storage, the energy barriers and rate constants in the range of 298–1000 K of reactions R1–R6-3 were calculated. The rate constants, fitted parameters of the rate coefficients, and energy barriers for different reactions are shown in Figure 21 and Figure 22 and Table 2.

The reaction rate constants of different reactions increased with increased reaction temperature (Figure 21). For each aging reaction of binder and curing agent, the reaction rate constant at the same temperature was basically proportional to the size of the energy barrier required for the reaction. For the aging reactions of NEPE propellant binder, the energy barriers of H abstraction reaction (R3-1 and R3-2) and oxidation (R4) were in the range of 79.3–91.2 kJ·mol^−1^, which were much smaller than those required for decomposition and nitration reactions (250.6–279.3 kJ·mol^−1^), indicating that H abstraction and oxidation reactions were more likely to occur than decomposition and nitration reactions. The decomposition of plasticizers and oxidizers in NEPE propellants had a strong influence on the binder aging reaction. The H abstraction and oxidation reactions initiated by decomposition products from plasticizers and oxidizers were the main types of reactions that occurred in propellant binders. As aging time increased, crosslinking reactions without potential energy barriers (R5-1 to R5-3) occurred between the products of H abstraction and oxidation reactions. This was also the main reason for the slightly increased trend for the maximum tensile strength of NEPE propellant in the middle of aging [18,36]. For the aging reaction of curing agent, the energy barriers required for three decomposition reactions of the agent were all greater than those required for binder aging reactions, indicating that the aging reaction of the curing agent was more difficult to occur, and occurred mainly in the later stages of propellant aging. Through calculations of the enthalpy and Gibbs free energy of the three possible degradation reactions speculated here, the direct decomposition of PT into PEG and TDI was seen to be less likely to occur. Although the reorganization reaction of amino radicals with alkyl radicals had a large reaction energy barrier for alcohol formation, it was thermodynamically exothermic and could proceed spontaneously.

## 3. Methods of Simulation and Calculation

### 3.1. Quantum Chemistry Simulation Methods

**Figure 23:**
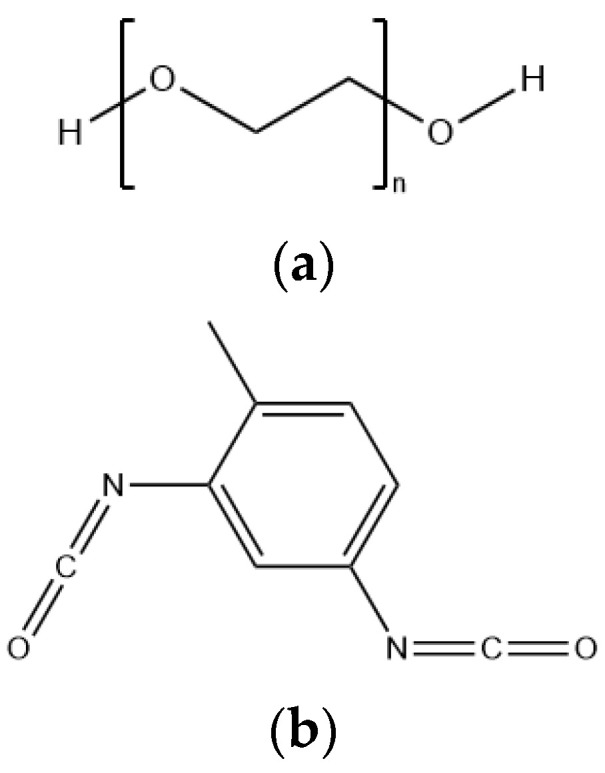
Structural formulae of (**a**) PEG and (**b**) TDI.

The molecular structures required for the simulation were constructed in GaussView, and different molecular structures were optimized at the B3LYP/6-311G(d,p) level, so that the molecular models with the lowest energy were obtained after frequency analysis to confirm that there was no imaginary frequency (IF). Then, the transition state (TS) involved in the reaction was calculated at the same level, and the intrinsic reaction coordinate (IRC) of the transition state was simulated to ensure the authenticity of the transition state in the reaction. Quantum chemical simulations were all calculated using the Gaussian program. The structural formulae of PEG and TDI are shown in Figure 23.

### 3.2. Calculation of Chemical Reaction Rate Constants

The reaction rate constant *k^CVT^* was calculated using the variational transition state theory of the CVT method. The calculation method of *k^CVT^* is represented by Formula (1):

(1) $$k^{CVT}\left(T\right)=\min\left\{\frac{\sigma k_{B}T}{h}\frac{\;Q^{GT}\left(T,s\right)}{Q_{R}\left(T\right)\;}\exp\left[-V_{MEP}\left(s\right)/\left(k_{B}T\right)\right]\right\}$$

where *σ* is the degeneracy of the reaction path and its value is the ratio of the number of rotational symmetries of the reactants to the transition state; *k_B_* is Boltzmann’s constant, J·K^−1^; *T* is the temperature; *h* is Planck’s constant, 6.626 × 10^−34^ J·s; *Q^GT^(T,s)* and *Q_R_(T)* are the partition functions of the transition state and the reactants at the reaction coordinate(s) at the temperature T, respectively; *V_EMP_* is the value of potential energy along the minimum energy path. Rate constants were all calculated using the Kisthelp software.

## 4. Conclusions

(1) The results of transition state calculations of aging reactions of NEPE propellant binder showed that the reactions could be divided into five types: decomposition, nitration, H abstraction, oxidation, and crosslinking reactions. Among them, H abstraction and oxidation reactions under the action of NO_2_ and O_2_ had lower energy barrier and faster reaction rate, which were the main reactions for aging of binder. The bond energy of C-O in the binder molecule was high and, thus, more difficult for homolytic reactions to occur, resulting in a higher reaction energy barrier and slower reaction rate for decomposition and nitration reactions. This was a secondary aging reaction mode for the binder. As the H abstraction and oxidation products contained alkyl and alkoxy radicals, respectively, their products could not exist stably and were prone to crosslinking reactions without potential barriers and driven by electronic effects, which increased the molecular weight of the binder and formed a network structure.
(2) For NEPE propellant curing agent aging reactions, transition state calculations showed that the main cause of curing agent aging was C(O)-N and C-O breakage and, after homolysis, active free radicals (NHC(O)O· and RNH·) and alcohols generated, and CO_2_ released at the same time. All three curing agent aging reactions damaged the long chain structure of the matrix to some extent and caused the gradual appearance of local holes and microcracks in the matrix. Compared with the binder aging reactions, the three curing agent reactions had higher energy barriers and, thus, were more difficult to react.
(3) Calculations of the energy barriers and rate constants of different aging reactions of NEPE propellant matrix showed that the aging of the matrix could be divided into two stages: in the first stage, H abstraction and oxidation reactions of long chain binder molecules occurred under the action of NO_2_ and O_2_, and active radicals generated by H abstraction and oxidation reactions easily combined with each other to produce crosslinking reactions, resulting in a sharp increase in binder molecular weight and an upward trend in the maximum tensile strength. This aging process mainly occurred in the middle stage of propellant aging, when most of the antioxidants had been consumed and the products of plasticizers and oxidizers started to gather in large quantities. In the second stage, the decomposition reaction of binder and curing agent occurred, which, on one hand, destroyed the curing network and crosslinking network of the matrix, and on the other hand, CO_2_ released by the decomposition reaction caused a porous structure and holes in the matrix during the process of diffusion and aggregation in the matrix. Under the joint action of both factors, the mechanical properties of NEPE propellant matrix showed a significant decline in the late aging stage.

To summarize, the future aging protection of the NEPE propellant matrix can be carried out in two ways: on one hand, through the design and addition of new antioxidants and stabilizers to reduce the impact of plasticizers and oxidizers on matrix aging, thus eliminating the formations of active free radicals in the product and delaying the first stage of matrix aging; on the other hand, the decomposition rate of the matrix could be minimized by monitoring the amount of CO_2_ gas released and reasonably adjusting storage conditions to delay the second stage of matrix aging. In following research, it is necessary to conduct relevant experiments to investigate the connection between the amount of CO_2_ gas release and the trends of matrix mechanical property decline during the aging process and to establish a more convenient and efficient means for monitoring matrix aging.

## Figures and Tables

**Table 1:** Homolytic dissociation energy of PT characteristic chemical bonds.

	Ⅰ	Ⅰ′	Ⅱ	Ⅱ′
BDE/a.u.	0.1072	0.0664	0.0635	0.0640

**Table 2:** Fitted parameters of the rate coefficients expressed in Arrhenius formula (k = AT^n^exp(−E_a_/RT), R = 1.988 × 10^−3^ kcal·mol ^−1^·K^−1^).

Channel	A	*n*	Ea
R1	4.56 × 10^11^	0.2081	255.41
R2	1.10 × 10^−24^	3.307	231.77
R3-1	4.41 × 10^−23^	3.205	69.08
R3-2	3.37 × 10^−3^	−4.137	72.86
R4	4.05 × 10^−7^	−2.436	71.52
R6-1	2.52 × 10^8^	0.9143	247.13
R6-2	2.64 × 10^11^	−0.2207	200.52
R6-3	7.78 × 10^13^	−0.7818	161.56

## Data Availability

Not applicable.

## Sample Availability

Samples of the compounds are not available from the authors.
